# Acrothoracican bioerosion as evidence of Early Cretaceous barnacles (Cirripedia) in northwestern Gondwana

**DOI:** 10.7717/peerj.21433

**Published:** 2026-06-15

**Authors:** Edwin-Alberto Cadena

**Affiliations:** 1 Escuela de Ciencias e Ingeniería, Grupo de Paleontología Neotropical Tradicional y Molecular (PaleoNeo), Universidad del Rosario, Bogotá, Colombia; 2 Smithsonian Tropical Research Institute, Panama City, Panama; 3 Field Museum of Natural History, Chicago, USA

**Keywords:** Ichnofossils, Valaginian, South America, Rosa blanca formation, Colombia

## Abstract

Ichnofossils preserved in hard substrates are sometimes the only evidence documenting the presence of certain groups of organisms within past ecosystems. The Early Cretaceous marine ecosystems of northwestern Gondwana (present-day Colombia) were highly diverse, comprising vertebrates, invertebrates and microorganisms. Despite this paleobiodiversity, no fossils or ichnofossils of barnacles (Cirripedia) have previously been reported from this region of South America. Here, I describe the first record of barnacle sclerobionts from the Early Cretaceous (Valanginian) of northwestern Gondwana, represented by 132 borings tentatively assigned to cf. *Rogerella*, preserved on a complete shell of the gryphaeid *Ceratostreon boussingaulti*, from the Rosa Blanca Formation in Zapatoca, Colombia. The borings exhibit an inverted, inequilateral pyramidal chamber in three dimensions, triangular in transverse section, with the deepest point located in the posterior part of the chamber. This discovery underscores the significance of detailed ichnological analyses for reconstructing the complexity of ancient benthic interactions and elucidating the ecological strategies of sessile suspension feeders through geologic time.

## Introduction

Borings are trace fossils produced by bioeroding organisms on hard substrates (skeletons, shells, wood, and rocks) (Taylor & Wilson, 2003; Wilson, 2007; Bertling et al., 2022; Dworczak et al., 2022). Among the sclerobionts organisms that produce borings on hard calcareous substrates are the acrothoracican cirripeds (barnacles) (de Saint-Seine, 1955; Donovan & Jagt, 2013; Kočová Veselská et al., 2021; Dworczak et al., 2022; Burger, Dimitrijević & Kiessling, 2024). The record of borings produced by acrothoracican spans from the Late Ordovician to the present (de Saint-Seine, 1955; Breton, 2011; Wisshak, Knaust & Bertling, 2019) and is distinct by creating small cavities that are generally pouch- or sac-shaped, which in many ichnospecies has a partial calcitic lining, and a comma-like opening (Seilacher, 1969; Tomlinson, 1969; Bromley, 2004). One of the most found fossil ichnogenera of acrothoracican barnacles is *Rogerella* de Saint-Seine, 1951: known from at least 14 ichnospecies (Wisshak, Knaust & Bertling, 2019). This ichnogenus is mostly known from Europe, North America and North Africa (de Saint-Seine, 1955; Zatoń, Wilson & Zavar, 2011; Donovan & Jagt, 2013; Collins, Donovan & Mellish, 2014; Demircan, Sorogy & Alharbi, 2021; Wisshak, Knaust & Bertling, 2019; Dworczak et al., 2022; El-Issawi et al., 2025, references therein), with few records from South America, including Brazil (Almeida, 2006) and Argentina (Brezina, Romero & Casadío, 2017).

Early Cretaceous marine ecosystems of northern Gondwana are particularly well documented in two regions of Colombia. One of these is the Alto Ricaurte Marine Reptile Lagerstätte (Barremian–Aptian), which is included in the list of important Geological Heritage Sites of the (IUGS (International Union of Geological Sciences), 2022). This area preserves an abundant and diverse fossil record within the La Paja Formation (Noè & Gómez-Pérez, 2020, and references therein), including marine reptiles, a dinosaur, invertebrates, and plants. The second region is Zapatoca, which preserves abundant fossil record from the Valanginian to Hauterivian Rosa Blanca Formation (shallow marine deposits), including marine reptiles, pterosaurs, invertebrates (bivalves, gastropods, brachiopods, crustaceans, among others), ichnofossils, microfossils and plant remains (Cadena & Gaffney, 2005; Luque, 2015; Benavides-Cabra & Páramo-Fonseca, 2020; Cadena, Unwin & Martill, 2020; Carrillo-Briceño & Cadena, 2022; Cadena & Combita-Romero, 2023; García-Guerrero et al., 2025; references therein). In terms of crustaceans, the Early Cretaceous record in northern Gondwana is represented primarly by crabs and lobsters (Luque, 2015; Luque et al., 2019; Gómez-Cruz, Bermúdez & Vega, 2015; González-León et al., 2019).

Here, I report the first evidence of acrothoracican barnacle borings for the northwestern part of Gondwana, preserved on the shell surface of an oyster identified as *Ceratostreon boussingaulti* d’Orbigny, 1842, from the Carrizal Member of the Rosa Blanca Formation in Zapatoca, Colombia. I discuss the palaeoecological implications of this finding and tentatively assign the borings to cf. *Rogerella*.

## Materials and Methods

### Fossil material

The shell of *Ceratostreon boussingaulti*, catalogued as UR-CP-0585, is housed in the Paleontological Collection of the Escuela de Ciencias e Ingeniería, Universidad del Rosario, Bogotá, Colombia. Approval for fieldwork and fossil-collecting activities was granted by the Servicio Geológico Colombiano (notification No. SGC-2-2024-005960). Specimen UR-CP-0585 corresponds to an articulated oyster preserving both left and right valves. It was collected *in situ* from a rock layer composed largely of a dense accumulation of *C. boussingaulti* individuals of various sizes, indicating a complete community encompassing multiple ichnogenetic stages However, despite intensive sampling of this layer and examination of additional specimens, UR-CP-0585 is the only specimen identified to date that exhibits borings.

The taxonomy of *Ceratostreon boussingaulti* remains contentious, with some authors and databases treating it as *Exogyra boussingaulti* (*e.g*., MolluscaBase, 2026), whereas others assing it to *Amphidonte (Ceratostreon) boussingaulti* (Malchus, 1990). Although resolving this taxonomic issue is beyond the scope of the present study, I reatin the usage of *Ceratostreon boussingaulti*, as adopted in the literature on the Rosa Blanca Formation (Etayo-Serna & Guzmán-Ospitia, 2019, and references therein) and in other studies (*e.g*., Kosenko & Metelkin, 2022).

### Photography, visualization and measurements

Specimen UR-CP-0585 was photographed and examined using a Nikon Eclipse SMZ1270 stereomicroscope at the laboratory of the Traditional and Molecular Neotropical Paleontology Group (PaleoNeo) at Universidad del Rosario, Bogotá, Colombia. To enhance the visualization of borings, a color depth map was generated using ImageJ 1.53 k software (Schroeder et al., 2021). Two close-up images were first transformed into 8-bit grayscale. Using the 3D plugin and the interactive 3D plot, a color depth map was generated using the spectrum LUT option. The length and width of 26 of the borings exhibited by UR-CP-0585 specimen were obtained using the cell analysis tool in ImageJ 1.53 k software (Schroeder et al., 2021). The results obtained for UR-CP-0585 were compared with published data for ichnospecies of *Rogerella* and additional ichnogenera reported in Dworczak et al. (2022). Following Dworczak et al. (2022), a principal component analysis (PCA) was conducted in R version 4.5.3 software (R Core Team, 2021) to explore the relationship between boring length and depth, and to assess whether the UR-CP-0585 specimen can be considered a new ichnospecies (File S1).

### Computer tomography (CT-scanning)

A general CT scan and 3D model of UR-CP-0585 specimen were obtained using a Carestream Dental Cone Beam at the Dental Image Laboratory in Bogotá, Colombia using the following parameters: maxillary option, 150 microns voxel size, 90 kV and 3.20 mA. A higher resolution Micro CT scan of the region having the borings was performed using a NSI X25 Micro-CT at the Field Museum of Chicago, RRID: SCR_026255 using the following parameters: voltage 130 kV, current 230 µA, voxel size 16.567 µm. Segmentations and measurements of borings depth were performed using 3D Slicer v5.8.1 (Fedorov et al., 2012). The CT data of the oyster having the barnacle borings (MorphoSource media ID 000790704) is available at https://www.morphosource.org/concern/media/000790704?locale=en.

### Artificial intelligence (AI) use

The Paperpal AI tool was used as an English grammar and style corrector, using the following prompt: “Correct the English grammar, style, and coherence of the following text, considering that it is part of a scientific article in paleontology.” Subsequently, the output was verified, modified, or retained according to the author’s criteria.

### Institutional abbreviation

UR-CP, paleontological collection, Facultad de Ciencias Naturales, Universidad del Rosario, Bogotá, Colombia.

## Geological and stratigraphic framework

**Figure 1:**
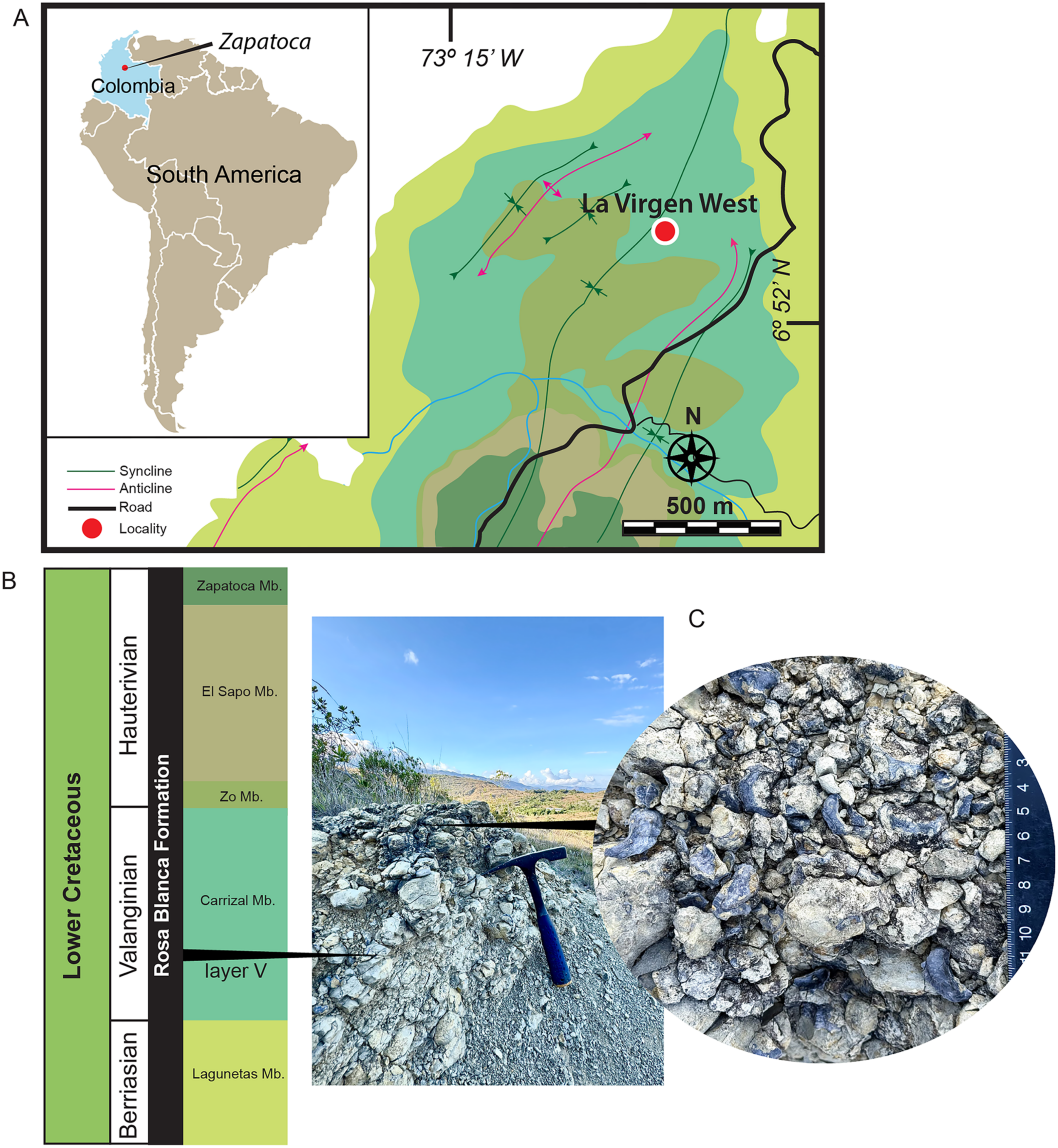
Location, geology, and stratigraphy. (A) Map of South America indicating the location of Zapatoca and the geology of the La Virgen West locality. (B) Stratigraphic column of the Rosa Blanca Formation, including its members and indicating layer V (following Etayo-Serna and Gúzman-Ospitia, 2019). (C) Photographs of layer V, where *Ceratostreon boussingaulti* bearing UR-CP-0585 cf. *Rogerella* was collected *in situ*.

The Rosa Blanca Formation represents a sabkha to subtidal-shallow marine depositional sequence composed of black to greenish-gray limestones, with intercalations of laminated calcareous shales and thick mudstones and wackestones (Morales, 1958; Moreno, 1990; Etayo-Serna & Guzmán-Ospitia, 2019). The formation is subdivided into five members, from base to top: Lagunetas, Carrizal, Zo, El Sapo, and Zapatoca (Etayo-Serna & Guzmán-Ospitia, 2019) (Figs. 1A, 1B). The Carrizal and El Sapo members are the most fossiliferous, yielding abundant vertebrate, invertebrate, and microfossil remains.

The fossil oyster bearing borings (specimen UR-CP-0585) was collected from the Carrizal Member, at the top of horizon V of the Rosa Blanca Formation, following the stratigraphic framework of Etayo-Serna & Guzmán-Ospitia (2019). Horizon V corresponds to a wackestone layer containing abundant specimens of *Ceratostreon boussingaulti* of various sizes forming an accumulation bank of approximately 50 cm (Fig. 1C). This layer also has gastropods, ammonoids, echinoids, other group of bivalves, and isolated teeth of pycnodontiform fishes (E-A Cadena, 2023, personal observations).

## Results

### Systematic ichnopaleontology

Ichnofamily Rogerellidae Codez and de Saint Seine, 1958

Ichnogenus cf. *Rogerella* de Saint-Seine, 1951

(Figs. 2, 3)

**Figure 2:**
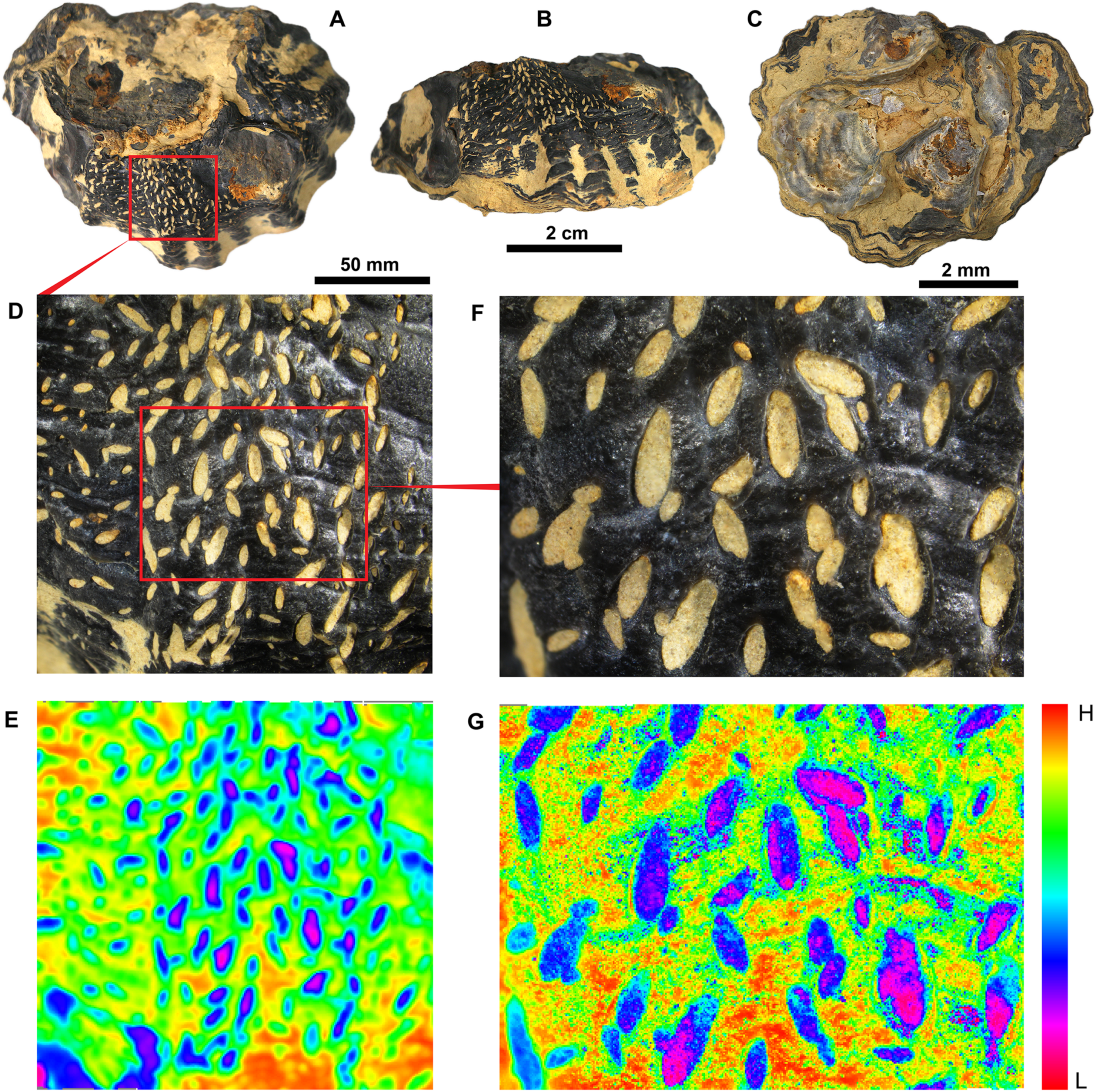
Shell of the gryphaeid *Ceratostreon boussingaulti*. (A) Left valve in ventral view. (B) Left valve in anterior view. (C) Right valve and umbo in dorsal view. (D, E) Close-up views of the borings of UR-CP-0585 cf. *Rogerella*. (E, F) Color maps of the borings of UR-CP-0585 cf. *Rogerella* showing the depth of the chambers. Abbreviations: H, high; L, low. Scale bar applies for (A)–(C).

**Figure 3:**
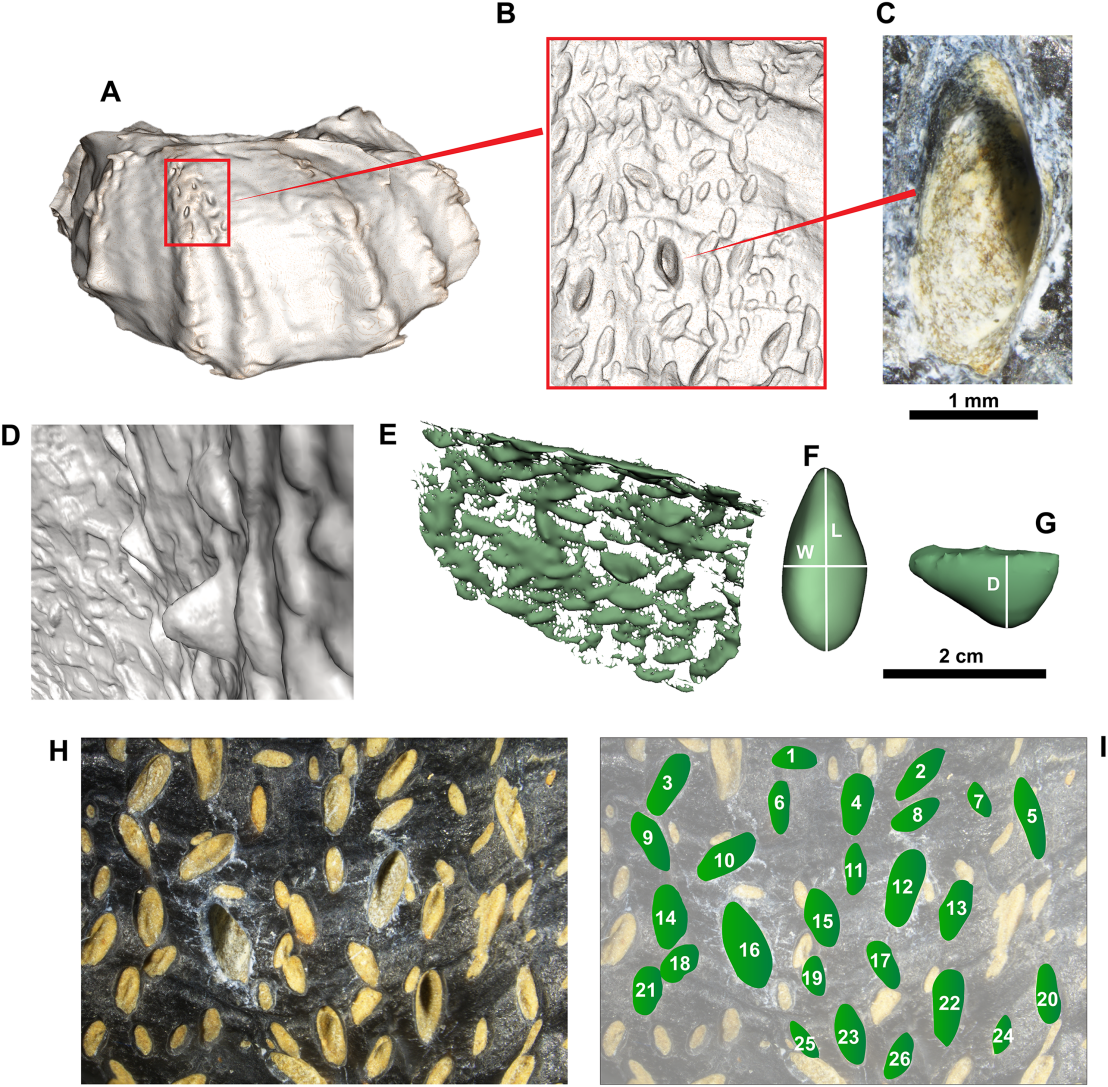
Details and 3D volume reconstruction of UR-CP-0585 cf. *Rogerella* borings. (A) Three-dimensional model of the shell of the gryphaeid *Ceratostreon boussingaulti* in ventral view, red rectangle indicates the area with the highest concentration of borings. (B, C) Close-up of the borings showing their overlap, close proximity, and the largest boring observed. (D, E) Micro-CT internal view and volume reconstruction of the chambers of cf. *Rogerella* (MorphoSource Media 000790704) with institutional catalog number UR-CP-0585 and DOI: [Insert DOI Here]. (F) Micro-CT reconstruction of the largest of the borings of UR-CP-0585 cf. *Rogerella* in apertural view. (G) Micro-CT reconstruction of the largest of the borings of UR-CP-0585 cf. *Rogerella* in transverse view. (H, I) Close-up of the borings of UR-CP-0585 cf. *Rogerella* indicating the numbering of at least 26 borings for which the length and width of the aperture were measured (see File S1). Abbreviations: D, depth; L, length; W, width. Scale bar 2 cm applies to (H) and (I).

Referred material.—UR-CP-0585, preserving 132 borings of different sizes distributed on the external surface and along the ribs of the larger valve of a gryphaeid oyster *Ceratostreon boussingaulti* d’Orbigny, 1842 (Fig. 2A).

Type locality and age.—La Virgen West locality, 6°52′24.61″N, 73°14′18.35″W (Fig. 1). The Carrizal Member has been assigned to the upper Valanginian (Lower Cretaceous) based on the ammonoid biostratigraphy zonation (Etayo-Serna & Guzmán-Ospitia, 2019; Szives et al., 2024), particularly by the occurrence of *Saynoceras verrucosum* within the member.

**Figure 4:**
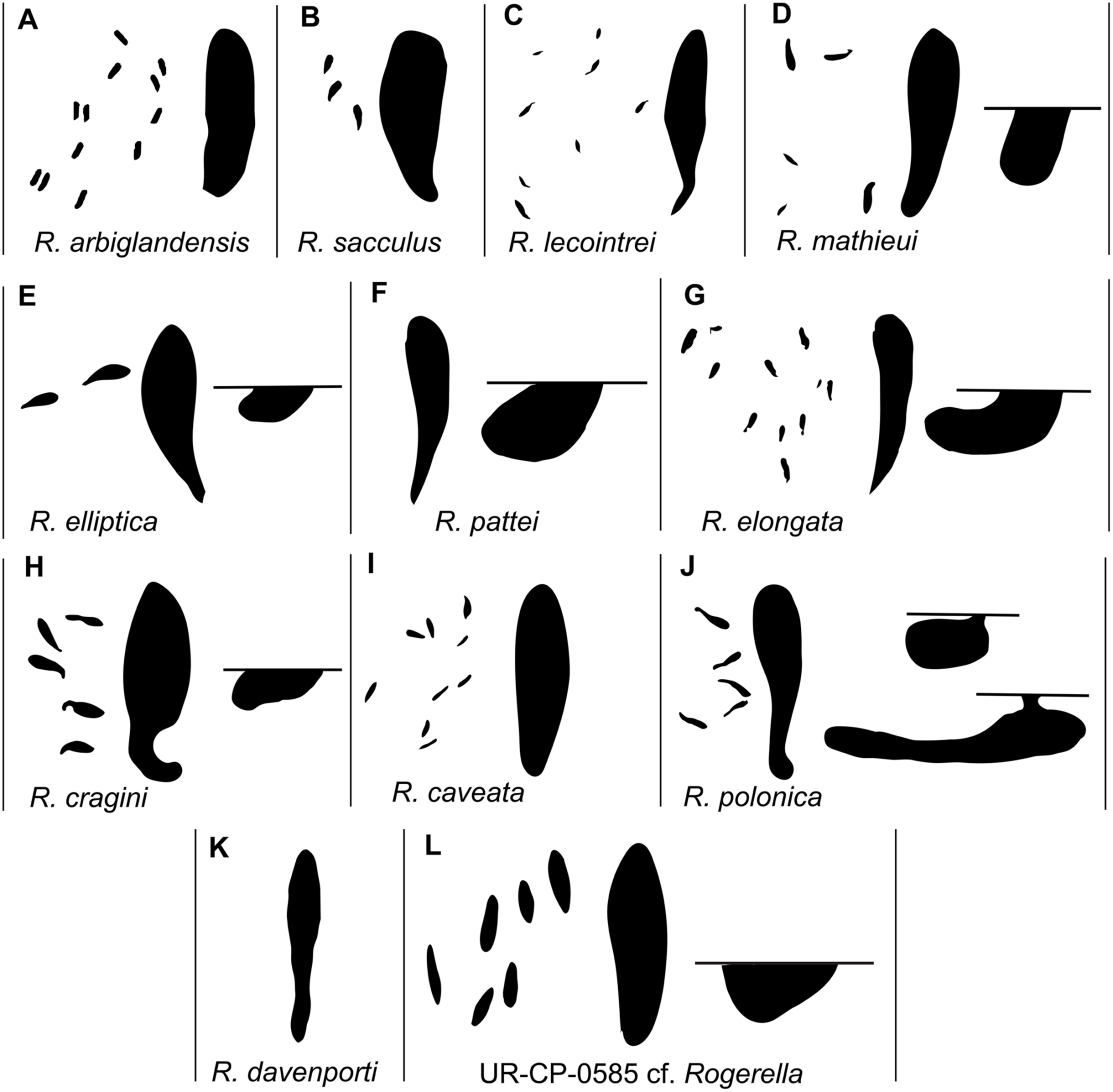
Comparisons of the aperture and chamber shape among ichnospecies of *Rogerella*. (A) *Rogerella arbiglandensi* (Smith, 1910; Wisshak, Knaust & Bertling, 2019). (B) *R. sacculus* (Mägdefrau, 1937; Wisshak, Knaust & Bertling, 2019). (C) *R. lecointrei* de Saint-Seine, 1951. (D) *R. mathieui* de Saint-Seine, 1955. (E) *R. elliptica* (Codez & de Saint-Seine, 1958; Wisshak, Knaust & Bertling, 2019). (F) *R. pattei* (de Saint-Seine, 1955; Wisshak, Knaust & Bertling, 2019). (G) *R. elongata* (Codez & de Saint-Seine, 1958; Wisshak, Knaust & Bertling, 2019). (H) *R. cragini* Schlaudt & Young, 1960. (I) *R. caveata* Tomlinson, 1963. (J) *R. polonica* (Bałuk & Radwański, 1991; Wisshak, Knaust & Bertling, 2019). (K) *R. davenporti* (Tomlinson, 1969; Wisshak, Knaust & Bertling, 2019). (L) UR-CP-0585 cf. *Rogerella* (this study). On the left side of each ichnospecies figure, several borings are redrawn from the original sources, whereas on the right side an enlarged outline of a single boring is shown in apertural view. For some ichnospecies, the chamber shape in transverse section is illustrated on the far right, mainly redrawn from Codez & de Saint-Seine (1958) and Schlaudt & Young (1960).

*Remarks*.—The borings of specimen UR-CP-0585 are assigned to the cf. *Rogerella* to the ichnogenus *Rogerella* based on the presence of pouch- or sac-shaped borings, consistent with the diagnosis of the genus presented by de Saint-Seine (1951). An additional diagnostic feature is the aperture, which typically exhibits one end wider than the other; moreover, some borings show a slightly curved terminal end. The characteristic comma-like shape of the aperture, regarded as a distinctive feature of the ichnogenus, may vary within the same specimen and is not always preserved in all borings, as observed, for example, in *R. cragini* (Fig. 4H).

The borings of UR-CP-0585 cf. *Rogerella* differ from those of other ichnospecies of *Rogerella* in exhibiting an inverted, inequilateral pyramidal chamber in three dimensions, triangular in transverse section, with the deepest point located in the posterior part of the chamber, as well as in the lack of clear peduncular slits in almost all borings. Despite these differences, I refrain from erecting a new ichnospecies or ichnogenus pending the discovery of additional specimens from the Rosa Blanca Formation, as well as a thorough and updated reexamination of the ichnogenus *Rogerella* and all ichnospecies attributed to it, and more broadly, all ichnogenera assigned to the Rogerellidae. Accordingly, I adopt open nomenclature and refer the material to cf. *Rogerella*, following the recommendations of Bengston (1988).

*Description*.—The borings of UR-CP-0585 cf. *Rogerella*, are preserved in a complete specimen of a gryphaeid specimen of *Ceratostreon boussingaulti* (Figs. 2A–2C, 3A). All borings are restricted to the external surface of the larger left valve, which also exhibits a slightly flattened attachment area interpreted as the site of substrate fixation and is entirely devoid of borings. The smaller right valve bears three attached juvenile individuals of *C. boussingaulti*, represented only by their right valves. The aperture of the boring is oval elongated-shaped and tapers toward the anterior end (Figs. 2D–2G, 3B, 3C, 3F).

In transverse section, the chambers are triangular, with the deepest point located in the posterior part of the chamber; in three dimensions, they formed inverted, inequilateral pyramids (Figs. 3D, 3E, 3G). Some borings exhibit a relatively wide, slightly curved anterior aperture (Figs. 3B, 3H, 3I). Aperture length ranges from 0.8 to 1.9 mm, and width from 0.3 to 0.9 mm (Figs. 3H, 3I, Table 1). The maximum preserved boring depth is 1.9 mm (Figs. 2E, 2G, 3C). The long axes of the borings are oriented approximately parallel to the ribs of the host shell, and a total of 132 borings occur on the external ventral surface of the oyster.

**Table 1:** Comparisons between different ichnospecies of *Rogerella*. Modified and updated from Tomlison, 1963.

Ichnospecies	Boring			Aperture		Form of aperture	Form of the chamber	Host	Age	Reference
	L	W	D	L	W					
*R. cragini*	≤1.9	≤0.7	0.9	0.8–1.9	0.3–0.7	One end widened and rounded, comma-like shape	Sac-shaped elongated towards one side	Gastropods	Middle Albian	Schlaudt & Young (1960)
*R. lecointrei*	–	–	–	0.5–3.1	0.6	Elongated oval, comma-like shape		Echinoids	Cretaceous	de Saint-Seine (1951)
*R. mathieui*	1.3–2.9	0.5–1.3	–	1.0–2.8	0.3–0.7	Sub-oval	Long sac-shaped with parallel sides, without lateral projection	Echinoids, Belemnites, Corals, Bivalves	Middle Jurassic, Upper Cretaceous, Miocene, Pliocene	Codez & de Saint-Seine (1958), Grygier and Newman, 1985
*R. polonica*	5.0–13.0	4.0–10.0	–	2.8–5.5	0.5–0.8	Elongated oval with a curved end	Wide and short sac-shaped, or very elongated tunnel. Very narrow exterior aperture	Gastropods	Middle Miocene	Bałuk & Radwański (1991)
*R. elongata*	2–4.5	0.5–1.1	2.6	1–2.3	0.3-	Elongated, with one end enlarged slightly	Sock-base shape, long elongation towards one side	Belemnites, Bivalves, Gastropods, Crinoids,	Triassic Jurassic	Codez & de Saint-Seine (1958), Wisshak, Knaust & Bertling (2019)
*R. elliptica*	1.1–2	0.4–0.9	1.1	0.9–1.8	0.3–0.5	Oval, with one end narrower	Small sac-shaped elongated towards one side	Belemnites, Bivalves	Cretaceous	Codez & de Saint-Seine (1958), Wisshak, Knaust & Bertling (2019)
*R. caveata*	11.3	5	2	0.3-3.4	0.1–1.4	Sub-oval		Bivalves	Pensylvanian, lowermost Cambrian	Tolimnson, 1963
UR-CP-0585 cf. *Rogerella*	≤1.9	≤1.1	1.2	0.8–1.9	0.3–0.9	Sub-oval	Inequilateral triangular shape	Bivalves (Gryphaeidae)	Valanginian	This study

Some of the larger borings are cross-cut by smaller ones, indicating multiple episodes of bioerosion, likely produced by the same taxon of barnacles. Most borings are filled with rock matrix, principally calcareous mud, and some exhibit slightly eroded lateral margins, partly due to the irregular surface of the host shell and possibly to minor dissolution effects.

*Comparisons*.—For comparisons, the shape of the aperture and the chambers for most of the ichnospecies of *Rogerella* listed by Wisshak, Knaust & Bertling (2019) were redrawn from the original descriptions and are presented in Figs. 4A–4L. Specimen UR-CP-0585 cf. *Rogerella*, most closely resembles *R. cragini* Schlaudt & Young, 1960 in the size of its aperture, which reaches a maximum length of 1.9 mm and a maximum width of 0.9 mm (Figs. 3H, 3I; 4L, File S1). The anterior end of the aperture in *R. cragini* exhibits a distinctly comma-like shape, which is the most pronounced among the ichnospecies of *Rogerella* (Fig. 4H).

In contrast, the apertures of the most complete borings of UR-CP-0585 cf. *Rogerella* are elongated- oval, with one end slightly wider than the other, resembling those of *R. sacculus* (Mägdefrau, 1937) (Fig. 4B) and *R. caveata* Tomlinson, 1963 (Fig. 4I). In *R. arbigladensis* Smith, 1910 (Fig. 4A), the aperture exhibits nearly equal widths at both ends and a shape that is more rectangular than oval. The aperture is elongated and narrow, and may exhibit one end slightly curved or comma-like in *R. lecointrei* de Saint-Seine, 1951 (Fig. 4C), *R. mathieui* de Saint-Seine, 1955 (Fig. 4D), *R. elliptica* Codez & de Saint-Seine, 1958 (Fig. 4E), *R. pattei* de Saint-Seine, 1955 (Fig. 4F), *R. elongata* Codez & de Saint-Seine, 1958 (Fig. 4G), and *R. polonica* Bałuk & Radwański, 1991 (Fig. 4J). Among the ichnospecies of *Rogerella*, *R. davenporti* Tomlinson, 1969 (Fig. 4K) exhibits the most elongated and narrow aperture.

In transverse section, the chamber of UR-CP-0585 cf. *Rogerella* borings exhibits an inequilateral triangular shape (Fig. 4L). This morphology differs from that of *R. elliptica*, *R. pattei*, and *R. cragini*, which display sac-shaped chambers elongated toward one side (Figs. 4E, 4H). The chamber of *R. mathieui* (Fig. 4D) is elongate and sac-shaped, with parallel sides and lacking any lateral projection. In *R. elongata* (Fig. 4G), the chamber exhibits a sock-like morphology with a pronounced elongation toward one side. In *R. polonica* (Fig. 4J), according to Bałuk & Radwański (1991), the chamber exhibits variable morphologies, ranging from wide and short sac-shaped forms to very elongated tunnels; however, all share the characteristic presence of a very narrow external aperture.

Variability in the morphology of the aperture and chamber among certain ichnospecies of *Rogerella* has been documented through both two and three-dimensional analyses (Schlaudt & Young, 1960, fig. 2; Dworczak et al., 2022, fig. 7). Nevertheless, the distinctly inverted, inequilateral pyramid configuration of the chamber in three dimensions, triangular in transverse section observed in UR-CP-0585 cf. *Rogerella* (Figs. 3D, 3E, 3G), appears to be unique among all described ichnospecies of *Rogerella*.

## Discussion

The occurrence of acrothoracican barnacle borings in a gryphaeid specimen of *Ceratostreon boussingaulti* from the Carrizal Member of the Rosa Blanca Formation represents the first ichnofossil record of acrothoracican barnacles from the northwestern region of Gondwana. The UR-CP-0585 cf. *Rogerella* specimen described herein expands the known paleogeographic distribution of *Rogerella* (de Saint-Seine, 1955; Almeida, 2006; Zatoń, Wilson & Zavar, 2011; Donovan & Jagt, 2013; Collins, Donovan & Mellish, 2014; Brezina, Romero & Casadío, 2017; Demircan, Sorogy & Alharbi, 2021; Wisshak, Knaust & Bertling, 2019; Dworczak et al., 2022; El-Issawi et al., 2025, references therein) and provides indirect but robust evidence for the presence of acrothoracican barnacles in Early Cretaceous (upper Valanginian) shallow-marine ecosystems of northern South America. This finding broadens the known crustacean record of these ecosystems, which previously included decapods such as crabs and lobsters (Luque, 2015; Luque et al., 2019; Gómez-Cruz, Bermúdez & Vega, 2015; González-León et al., 2019), by documenting the presence of barnacles through their boring traces.

With 132 borings on the external surface of the bivalve shell, specimen UR-CP-0585 cf. *Rogerella* represents the highest number of barnacle borings ever recorded on a single host substrate. This high degree of bioerosion indicates that gryphaeid oysters constituted favorable microhabitat for acrothoracican barnacles, as evidenced also in extant species (Zuschin & Baal, 2007). The size and thickness of the oyster shells likely contribute to their attractiveness as a habitat for barnacles (El-Hedeny & El-Sabbagh, 2007; Zatoń, Wilson & Zavar, 2011).

The occurrence of overlapping borings, in which smaller (younger) apertures truncate or penetrate larger (older) ones, indicates that the colonization of the bivalve shell occurred through multiple, temporally distinct episodes of bioerosion. This sequential pattern suggests a long-term interaction, in which successive generations of barnacles repeatedly exploited the same substrate, similar behavior has been documented in other barnacles and host substrates including bone of vertebrates (Gregg, 1948; Collareta et al., 2023). These successive episodes of bioerosion, together with the irregular morphology created by the valve ribs, likely account for the slight variation in boring shape, particularly among smaller specimens. However, most of the segmented borings (Figs. 3D, 3E) exhibit the diagnostic pattern described for UR-CP-0585 cf. *Rogerella*.

Micro-CT analysis of the borings in specimen UR-CP-0585 cf. *Rogerella* (Figs. 3D–3G) reveals that the inverted, inequilateral pyramidal shape of the chamber is shared by nearly all borings (Fig. 3D) and differs markedly from the morphotypes of acrothoracican borings described and illustrated by Dworczak et al. (2022, fig. 7).

**Figure 5:**
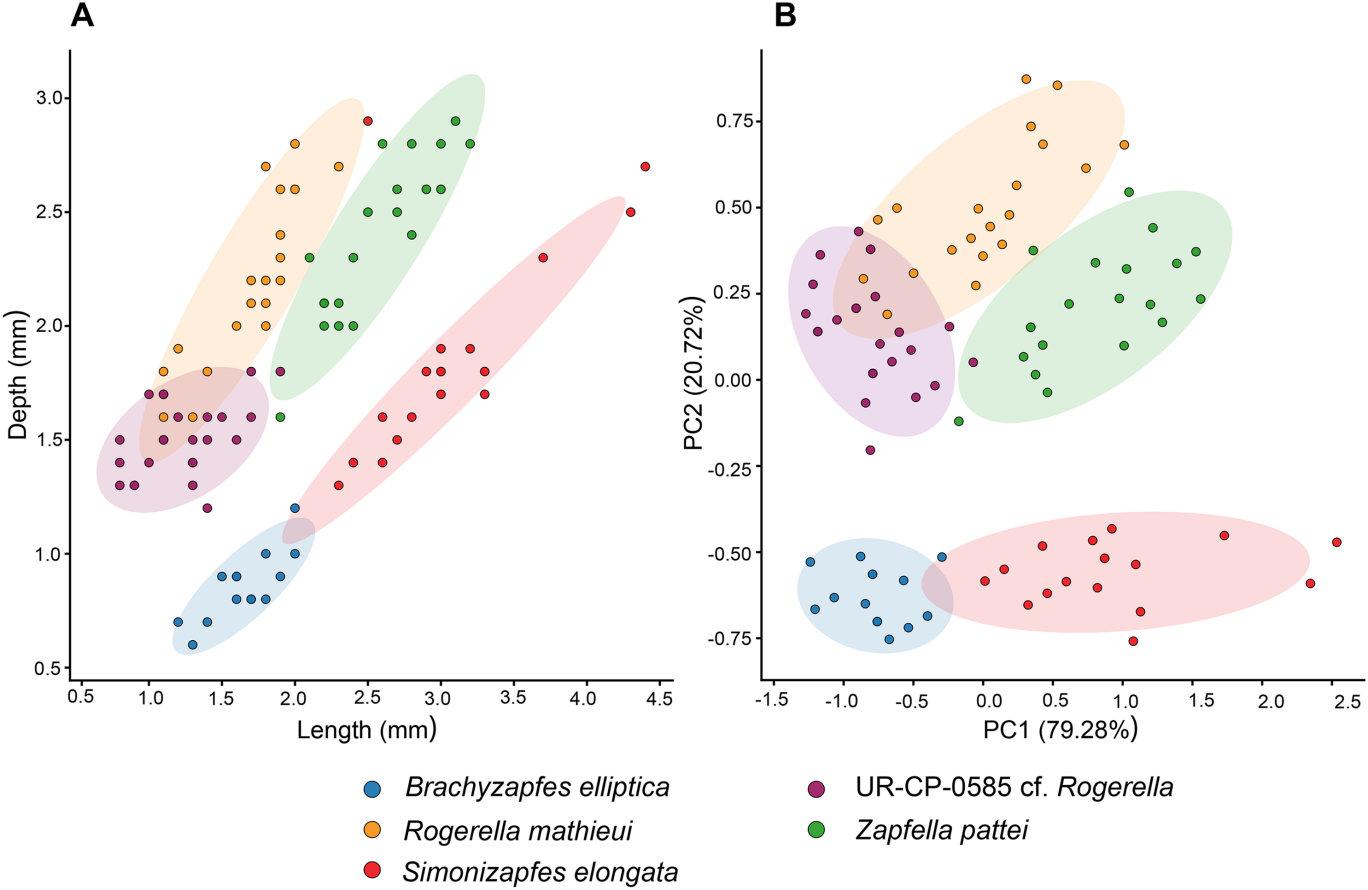
Correlation between length and depth of the borings of some acrothoracican. (A) Relationships between the aperture length and depth of acrothoracican borings based on Dworczak et al. (2022, appendix 3). (B) PCA of acrothoracican showing borings divided into five clusters, using the K-means algorithm. Ellipses mark 95% confidence intervals for each cluster.

The relationship between the length and depth of the borings shows a positive correlation, allowing each ichnotaxon to form clearly defined clusters, including UR-CP-0585 cf. *Rogerella* (Fig. 5A). Principal Component Analysis (PCA) indicates that PC1 (79.28%) reflects overall size variation, whereas PC2 (20.72%) captures shape differences, particularly the relative depth of the traces (Fig. 5B). K-means clustering supports the separation of ichnospecies into distinct morphospaces, with partial overlap between *Rogerella mathieui* and UR-CP-0585 cf. *Rogerella*, suggesting transitional morphologies or taxonomic uncertainty.

Comparisons among the different ichnospecies of *Rogerella* (Fig. 4) reveal a wide spectrum of variability in both the outline of the borings and the shape of the chamber or cavity. This variability warrants further detailed investigation, including quantitative measurements, micro-CT scanning, and direct examination of specimens, in order to refine the diagnosis of the ichnogenus *Rogerella* and to evaluate the potential subdivision of some ichnospecies into new ichnogenera.

## Conclusion

Specimen UR-CP-0585, described here from the upper Valanginian of Colombia, represents one of the highest numbers of barnacle borings cf. *Rogerella* recorded on a single host, in this case an oyster of *Ceratostreon boussingaulti*. Moreover, UR-CP-0585 cf. *Rogerella* demonstrates that barnacles were also a component of marine ecosystems in northwestern Gondwana during the Early Cretaceous, particularly during the upper Valanginian. This finding highlights the importance of detailed ichnological analyses for reconstructing the complexity of past benthic interactions and for understanding the ecological strategies of sessile suspension feeders through geologic time. The invertebrate, vertebrate, and plant remain from the Valanginian–Hauterivian of the Rosa Blanca Formation in Colombia constitute a unique opportunity for the continuation of future ichnotaxonomic and ichnological studies, which could help us better understand the ecological interactions among marine organisms during the Early Cretaceous.

## Supplemental Information

10.7717/peerj.21433/supp-1 Supplemental Information 1 Raw data measurements. Data of length, width, and depth for several ichnospecies

## Additional Information and Declarations

### Field Study Permissions

The following information was supplied relating to field study approvals (*i.e*., approving body and any reference numbers):

Approval for fieldwork and fossil digging was obtained from the Servicio Geológico Colombiano, notification [N° SGC-2-2024-005960].

### Data Availability

The following information was supplied regarding data availability:

The raw measurements of the borings are available in the Supplemental File.

The CT data is available at MorphoSource (ID: 000790704):

- https://doi.org/10.17602/M2/M790704.

- https://www.morphosource.org/concern/media/000790704?locale=en.

### New Species Registration

The following information was supplied regarding the registration of a newly described species:

LSID for the publication.

https://zoobank.org/References/0AAD2ED2-210F-4E63-9E62-DD604BF98DDC.

LSID for the taxonomic registration

https://zoobank.org/NomenclaturalActs/c113651f-cc77-41ac-8817-79873a42ad22.
